# Hydrogen Absorption Performance and O_2_ Poisoning Resistance of Pd/ZrCo Composite Film

**DOI:** 10.3390/ma16083159

**Published:** 2023-04-17

**Authors:** Yiyao Qian, Ruijun Qian, Hetian Feng, Dachuan Zhu, Chaoling Wu

**Affiliations:** College of Material Science and Engineering, Sichuan University, Chengdu 610064, China

**Keywords:** ZrCo film, Pd/ZrCo composite film, hydrogen absorption performances, O_2_ poisoning resistance

## Abstract

In order to enhance the hydrogen absorption performance and poisoning resistance of ZrCo to O_2_, Pd/ZrCo composite films were prepared by direct current magnetron sputtering. The results show that the initial hydrogen absorption rate of the Pd/ZrCo composite film increased significantly due to the catalytic effect of Pd compared with the ZrCo film. In addition, the hydrogen absorption properties of Pd/ZrCo and ZrCo were tested in poisoned hydrogen mixed with 1000 ppm O_2_ at 10–300 °C, where the Pd/ZrCo films maintained a better resistance to O_2_ poisoning below 100 °C. The mechanism of poisoning was investigated jointly by first-principles calculation combined with SEM-EDS elemental mapping tests. It is shown that the poisoned Pd layer maintained the ability to promote the decomposition of H_2_ into hydrogen atoms and their rapid transfer to ZrCo.

## 1. Introduction

In recent years, nuclear fusion energy, regarded as a principal source for the future, has been extensively researched based on the International Thermonuclear Experimental Reactor (ITER) project due to the fact of its advantages of abundant fuel resources, high energy density, and low environmental pollution [1,2,3,4]. Since radioactive gaseous tritium is used as the raw gas for nuclear fusion energy and needs to be safely stored and recovered, hydrogen isotope storage materials are a crucial part of the ITER project. As a typical hydrogen storage alloy, ZrCo alloy has attracted the attention of researchers, which avoids the disadvantages of radiation and spontaneous combustion while maintaining good hydrogenation/dehydrogenation properties, similar thermodynamic properties, and excellent helium-trapping ability compared with U [5,6]. However, ZrCo alloy still suffers from some shortcomings, such as poor initial hydrogen absorption kinetics, which can be improved by alloying modification and morphological control. Luo et al. added the Mo element to ZrCo alloys, where the initial hydrogen absorption time of the ZrCo_0.8_Mo_0.2_ sample was only one-third of that of ZrCo [7]. Yuan et al. reported a honeycomb ZrCo alloy with high porosity and specific surface by morphological control which accelerated the initial hydrogen absorption kinetics [8]. In addition, trace impurity gases, such as O_2_, N_2_, CO, and CO_2_, preferentially react with ZrCo alloy, leading to the destruction of the hydrogen storage capacity, which is commonly found in hydrogen isotope storage and delivery systems. Typically, surface modification is an effective way to improve the resistance of hydrogen storage alloys to gas impurities. By virtue of its high selectivity for hydrogen permeation, Pd alloy allows for normal hydrogen absorption while preventing the permeation of impurity gas [9,10]. Zhang et al. used electroless plating to prepare a Pd-Ag membrane on the surface of Zr-based alloys, which significantly elevated the poisoning resistance of alloy in H_2_ containing 1 vol.% air at different temperatures [11]. Nevertheless, Pd coated by electroless plating showed poor uniformity, with the microcracks and partial flaking of the membrane after hydrogenation [12].

In this work, based on the aforementioned information, we chose the magnetron sputtering method to deposit a series of Pd, ZrCo, and Pd/ZrCo films for their prominent hydrogen storage properties and poisoning resistance. Herein, the hydrogen absorption performance of ZrCo and Pd/ZrCo films in pure hydrogen or hydrogen atmosphere containing 1000 ppm O_2_ was compared and analyzed, and the first-principles calculation combined with the experimental results were applied to investigate the poisoning mechanism of trace O_2_ on the films.

## 2. Experimental

### 2.1. Material Preparation and Characterization

The Pd target (99.99%) and ZrCo target (99.9% Zr:Co = 1:1 at%) were used as the sputtering source to deposit the Pd, ZrCo, and Pd/ZrCo films on the polished Si/SiO_2_ substrate via direct current magnetron sputtering. The detailed fabricating steps of the Pd and ZrCo films were as follows: First, the sputtering chamber was vacuumized and maintained at a basic pressure of 2 × 10^−3^ Pa and then heated to 150 °C to remove the adsorbed gas in the chamber, which would cause the oxidation of the film. Next, high-purity argon (99.999%) was used to adjust the working pressure of the chamber to 3 Pa, and 2500 V ion beam cleaning was performed to eliminate the surface oxide layer of the target. Finally, a series of ZrCo and Pd films were deposited under sputtering conditions with a target power of 200 W, working pressure of 0.9 Pa, and substrate temperature of 500 °C. The specific sputtering parameters are shown in the Table 1. Specifically, for the Pd/ZrCo composite film, the Pd film was deposited on the SiO_2_ substrates for 5 min, 10 min, or 20 min, and then the ZrCo film was deposited on the surface of the Pd film for 60 min. These three kinds of samples with different thicknesses of Pd layer were named as 5-Pd/ZrCo, 10-Pd/ZrCo, and 20-Pd/ZrCo. In addition, the substrate was rotated at a constant speed during the sputtering process so that the film was deposited uniformly.

The phases of the Pd, ZrCo, and Pd/ZrCo films were all performed by X-ray diffractometer (XRD-6000, SHIMADZU, Kyoto, Japan) with the Cu-Kα target in the 2θ range of 20°~70°. The microstructure of the surface and cross-section was characterized by scanning electron microscopy (SEM, JSM7500, Tokyo, Japan), and the corresponding elemental mapping was observed by energy-dispersive X-ray spectrometer (EDS) elemental mapping.

### 2.2. Hydrogen Storage Property Measurements

The hydrogen storage properties were tested using a Sievert’s-type volumetric apparatus (MH-PCT-100, GRIMAT, Beijing, China). Prior to testing, the quantitative samples needed to be activated. The detailed activation procedures are as follows: First, the samples were loaded into the reaction vessel (65 mL) of the MH-PCT-100, and then the reaction vessel was vacuumized by the mechanical pump and maintained at a basic pressure of less than 1 kPa. Next, the reaction vessel was heated to 400 °C and held for 2 h by the resistive heater. As the samples cooled down to room temperature, the hydrogen absorption kinetic measurements were performed in pure H_2_ at 0.5 MPa. The poisoned H_2_ mixed with 1000 ppm O_2_ was prepared using a dynamic volumetric instrument (LFX-3000, LAIFENGKEJI, Chengdu, China).

### 2.3. Computational Method

The calculations were performed using the Vienna ab initio simulation package (VASP) based on the spin-polarized density generalized function theory (DFT) [13,14,15]. The projective suffix plus plane wave (PAW) pseudopotential method under the generalized gradient approximation (GGA) was used, and the electronic interactions are described using the pseudopotentials with an energy cutoff of 400 eV [16,17].

A 3 × 3 supercell was used to simulate the ideal Pd (111) plane, a 3 × 2 supercell was used to simulate the ideal ZrCo (110), the bottom two atoms of the four-layer structure were fixed during the calculation to simulate the bulk phase, and the top two atoms were relaxed to simulate the surface. A vacuum space of 15 Å was introduced to avoid the interactions of the adjacent slabs. We performed Brillouin-zone integration using Monkhorst–Pack grids of special points [18]. The 4 × 4 × 1 *k*-point meshes were employed for the ZrCo (110) and Pd (111) plane. The convergence criterion of the force applied to the relaxation of each atomic structure was 0.01 eV/Å, and the energy convergence criterion of the self-consistent iteration was 10^−5^ eV.

The adsorption energy of O_2_ on all configurations were defined by the following formula [19]:

(1) $$∆E=E_{slab+O_{2}}-E_{slab}-E_{O_{2}}$$

where slab represents the supercell of Pd (111) or ZrCo (110), $E_{slab+O_{2}}$ is the total energy of the slab with adsorbed O_2_, $E_{slab}$ refers to the total energy of the clean slab, and $E_{O_{2}}$ denotes the total energy of the isolated O_2_ molecule.

## 3. Results and Discussion

### 3.1. Structural Characterization

**Figure 1:**
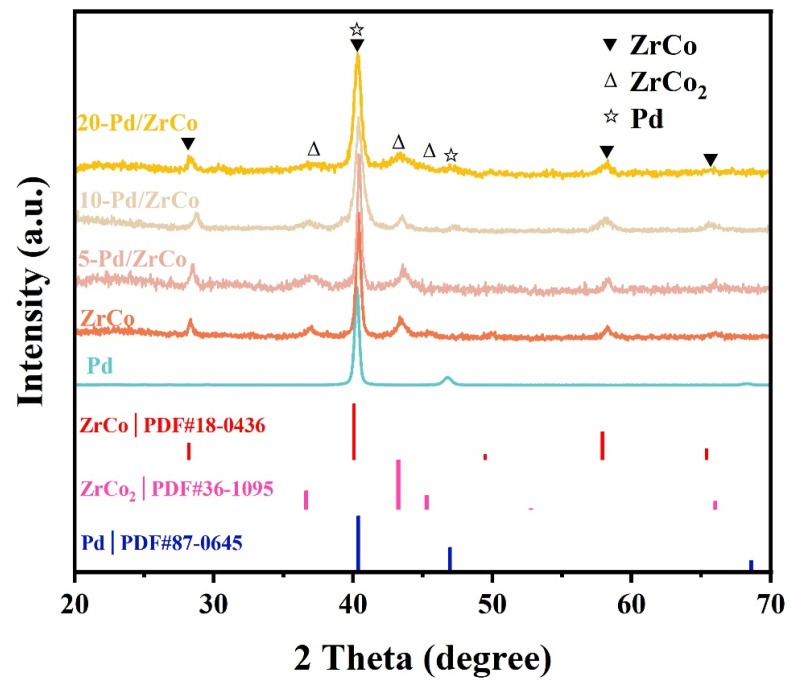
XRD patterns of the Pd, ZrCo, and Pd/ZrCo films.

The X-ray diffraction patterns of the Pd, ZrCo, and Pd/ZrCo films are depicted in Figure 1. It reveals that the Pd film obtained by the above magnetron sputtering method is a pure phase of Pd (PDF#87-0645). As for the ZrCo film, it is composed of a major phase of ZrCo (PDF#18-0436) with the space group of *Pm-3m* and a small amount of ZrCo_2_ secondary phase (PDF#36-1095), which is a stable phase formed in the cobalt-rich side [20]. Similar results were found in previous studies [21,22]. For the three Pd/ZrCo samples, the patterns of the 10-Pd/ZrCo and 20-Pd/ZrCo display an additional peak at approximately 2θ = 47.0°, and the pattern for 5-Pd/ZrCo remains almost unchanged compared with the pattern for the ZrCo films due to the very thin thickness of the Pd layer of 0.38 µm, while the 10-Pd/ZrCo and 20-Pd/ZrCo films have a thicker thicknesses of the Pd layer of 0.73 µm and 1.53 µm, respectively. In addition, the main peak of the Pd coincides with the main peak of the ZrCo at approximately 2θ = 40.4°, resulting in their patterns being relatively close.

**Figure 2:**
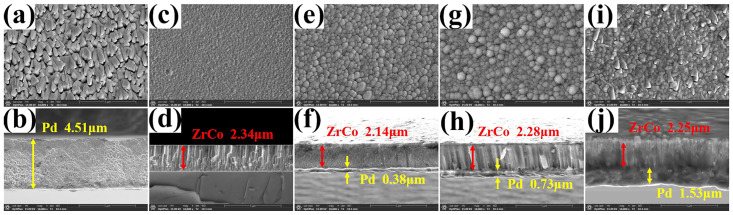
Surface and cross-sectional SEM pictures at a magnification of 10,000×: (**a**,**b**) Pd; (**c**,**d**) ZrCo; (**e**,**f**) 5-Pd/ZrCo; (**g**,**h**)10-Pd/ZrCo; (**i**,**j**) 20-Pd/ZrCo films.

Figure 2a–j display the surface and cross-section SEM images of the Pd, ZrCo, and Pd/ZrCo films at a magnification of 10,000×. From the surface morphology of the samples, the Pd film is composed of stacked cones particles, while the ZrCo film is composed of uniformly fine particles. As for the Pd/ZrCo composite films, the surface layer is composed of agglomerates consisting of cauliflower-like clusters due to the deposition of ZrCo particles along the conical Pd particles. When the deposition time of Pd is 20 min, the particles on the surface of the 20-Pd/ZrCo film grow up, and the uniformity and compactness decrease. Moreover, the cross-sectional morphology of each sample is also exhibited in Figure 2, and the thickness of the films is listed in Table 2. It is observed that the cross-section of the Pd layer is scaly, while the ZrCo layer is columnar. Meanwhile, the thickness of the Pd layers increase linearly with the increase in the Pd deposition time, and the average deposition rate is approximately 0.08 μm/min.

### 3.2. Hydrogenation Performance in Pure H_2_

**Figure 3:**
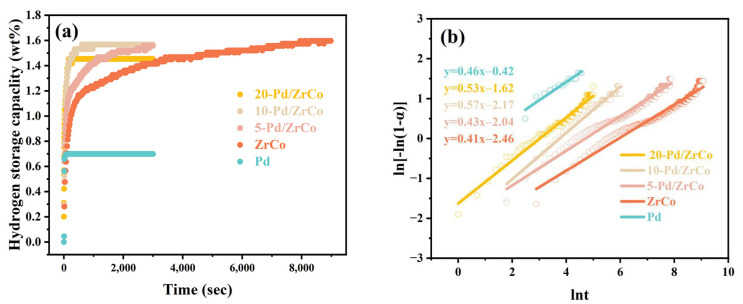
(**a**) The initial hydrogen absorption kinetic curves of the Pd, ZrCo, and Pd/ZrCo films under 0.5 MPa of H_2_ at room temperature; (**b**) plots of $ln\left[-ln\left(1-\alpha \right)\right]$ vs. lnt for the hydrogen absorption kinetic curves.

Figure 3a displays the initial hydrogen absorption kinetic curves of the Pd, ZrCo, and Pd/ZrCo films under 0.5 MPa of H_2_ at room temperature. It can be seen that the hydrogen absorption kinetics of the Pd/ZrCo films was greatly improved compared with that of the ZrCo film, which is ascribed to the addition of the Pd layer. The detailed hydrogen absorption capability and 95% saturated hydrogen absorption time of the samples are listed in Table 2. Generally, the hydrogen absorption of the alloy includes the following three processes: ① dissociation of hydrogen molecules into hydrogen atoms on the alloy surface; ② diffusion of hydrogen atoms into the alloy; and ③ reaction of hydrogen atoms with the alloy to form hydrides. Apparently, the hydrogenation rate depends on the combined effect of these processes [12,23]. As illustrated in Figure 3b, the hydrogenation rate of the pure Pd film was much higher than that of the ZrCo film, since Pd has excellent catalytic activity towards hydrogen molecules, which enables hydrogen molecules to dissociate into hydrogen atoms more quickly and greatly increases the hydrogenation rate. In this way, the construction of the Pd/ZrCo composite film urges one side of the sample to absorb hydrogen rapidly owing to its combination with Pd, while the other side absorbs hydrogen normally. As a result, the total hydrogenation rate of the Pd/ZrCo film is faster than that of the ZrCo film.

In order to study the hydrogenation kinetic mechanism of the Pd, ZrCo, and Pd/ZrCo films, the hydrogen absorption model Johnsone–Mehl–Avrami–Kolmogorov (JMAK) was used for the characterization, as follows [24]:

(2) $$ln\left[-ln\left(1-\alpha \right)\right]=\eta lnk+\eta lnt$$

where *t* is the reaction time, *k* denotes the hydrogenation reaction rate constant, α represents the ratio of the alloy to the hydride, and η is the Avrami exponent. Based on the data from the hydrogen absorption curve, the kinetic parameters *k* and *η* can be obtained by a linear fit of $ln\left[-ln\left(1-\alpha \right)\right]$ vs. lnt, as displayed in Figure 3b. According to Equation (2), ηlnk and η correspond to the intercept and slope of the fitted straight line. The final calculations of *k* are presented in Table 2, implying that the hydrogenation rate rises with the increase in the deposition time of the Pd layer. However, although the 20-Pd/ZrCo film exhibits a higher hydrogenation rate with a rate constant *k* that is nearly 16 times higher than that of the ZrCo film and twice that of 10-Pd/ZrCo film, the increase in the thickness of the Pd film obviously further reduces the saturated hydrogen absorption capacity of the sample. Therefore, the subsequent measurements were performed on the 10-Pd/ZrCo film.

### 3.3. Hydrogenation Performance against O_2_ Gaseous Impurity

**Figure 4:**
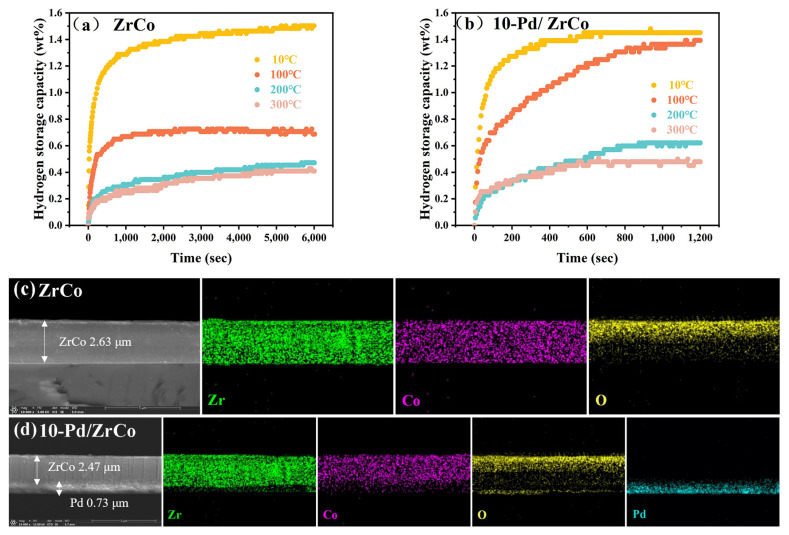
(**a**,**b**) Hydrogen absorption curves of ZrCo and 10-Pd/ZrCo after poisoning at 10 °C, 100 °C, 200 °C, and 300 °C; (**c**,**d**) SEM–EDS elemental mapping of the cross-sections of the ZrCo and 10-Pd/ZrCo films after poisoning at 100 °C.

For the ITER project, the hydrogenation performance of the hydrogen storage materials is easily deteriorated by the trace O_2_ impurity gas in the reactor during the system’s operation. To evaluate the poisoning influence of the O_2_ impurity on the ZrCo and 10-Pd/ZrCo films, the hydrogen absorption performance of the samples was tested in a hydrogen atmosphere containing 1000 ppm O_2_ at 10 °C, 100 °C, 200 °C, and 300 °C, as illustrated in Figure 4a,b, respectively. Meanwhile, in order to explore the distribution of the different elements within the films, the samples poisoned at 100 °C were characterized by SEM-EDS elemental mapping after the hydrogen absorption test, as displayed in Figure 4c,d.

It is observed that the hydrogen absorption capacity of the ZrCo film did not degrade obviously after poisoning with 0.5 MPa H_2_ (1000 ppm O_2_) at 10 °C; when the temperature rose to 100 °C, the film was poisoned obviously, and the saturated hydrogen absorption capability decreased from 1.50 wt% to 0.71 wt%; as the temperature came up to 200 °C, the O_2_ gas impurity led to the deterioration of the hydrogen storage capacity, and the level of poisoning further deepened, resulting in the saturated hydrogen absorption capacity dropping to 0.47 wt%; while the poisoning temperature continued to elevate to 300 °C, the degree of poisoning hardly changed compared with that at 200 °C.

**Figure 5:**
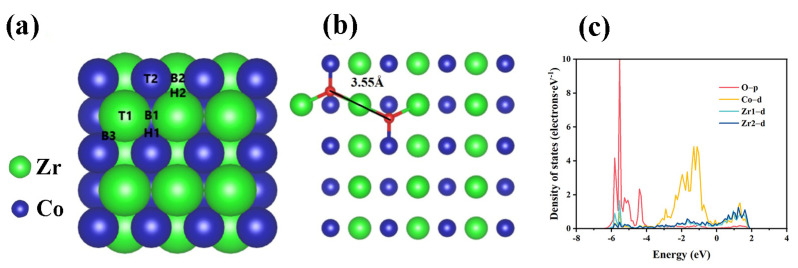
(**a**) Initial adsorption sites of the O_2_ molecules on ZrCo (110); (**b**) top view of the B3b site; (**c**) PDOS distribution of the B3b configuration.

The poisoning of the ZrCo by trace O_2_ in the mixture gas is discussed by theoretical calculation. In this work, the initial adsorption sites of O_2_ molecules on ZrCo (110) are displayed in Figure 5a. Three possible configurations exist for each position: ① perpendicular to the surface; ② molecular axis parallel to the surface; and ③ rotated by 90° based on the structure of ②. Thus, this implies that 21 adsorption states exist on bare ZrCo (110). Subsequently, for the purpose of obtaining the most stable adsorption site of O_2_ molecules on ZrCo (110), the adsorption energy and O-O bond length at each site were calculated according to Equation (1), as recorded in Table S1. As ∆E is negative, the greater the absolute value of the adsorption energy, the stronger the interaction between the ZrCo (110) and O_2_ molecules. According to the calculated results, the most stable adsorption site of O_2_ molecules on ZrCo (110) is at the position of B3b, as demonstrated in Figure 5b, where the adsorption energy of the O_2_ molecules is −7.46 eV, and the O-O bond length is 3.55 Å, which is approximately 2–3 times longer than the bond length of the isolated O_2_ molecules (1.2 Å). As a consequence, O_2_ is completely dissociated during adsorption on the ZrCo (110) owing to the huge adsorption energy and O-O bond length. To further analyze the atomic bonding of the ZrCo (110) and O atom, the partial density of states (PDOS) of a dissociated oxygen atom with the nearest two zirconium atom and a cobalt atom was calculated, as indicated in Figure 4c. After O adsorption on the ZrCo (110), the 4d orbit of the Zr atom and the 3d orbit of the Co atom in the zone above the Fermi energy level (0 eV) matches the 2p orbit of the O atom one by one. In the zone below the Fermi energy level, the 2p orbit of the O atom has clear resonance peaks with the 4d orbit of the Zr atom and the 3d orbit of the Co atom. The overlap of the density of states represents a contribution to the bonding, which indicates a strong interaction of ZrCo with the O atom.

The result is in agreement with the SEM–EDS elemental mapping expression of the cross-section. As seen in Figure 4c, a large number of O atoms gathered in the upper part of the ZrCo film, and the distribution of O gradually became sparse from the surface to the substrate. This means that a dense oxide layer (i.e., ZrO_2_) was generated on the surface of the ZrCo film after poisoning at 100 °C, as shown in the XRD patterns in Figure S1, and only a little ZrO_2_ was distributed in the interior of the film. Since Zr is a vital forming element of the hydride, the production of ZrO_2_ reduces the theoretical hydrogen absorption capacity of the ZrCo sample. Moreover, the ZrO_2_ oxide layer impedes the dissociation and diffusion of H_2_ molecules, leading to a lower hydrogenation reaction activity [25]. In addition, the hydrogen absorption capacity of the ZrCo sample in hydrogen atmosphere containing 1000 ppm O_2_ exhibited a dependence on the temperature, and the hydrogen absorption content decreased with the increase in the temperature. The temperature dependence of the diffusion coefficient of O could explain this trend [26]. When the temperature was 10 °C, the diffusion coefficient of O was low to form the ZrO_2_ oxide layer, and only small amounts of oxides could be generated on the surface of the ZrCo film, which makes it difficult to hinder hydrogen absorption. Accordingly, while the temperature exceeded 100 °C, the diffusion coefficient of O subsequently ascended. In this case, the O atoms diffused rapidly from the surface to the depth of the film thickness, forming a dense ZrO_2_ layer that hindered the hydrogen permeation. Therefore, the ZrCo films exhibited a strong poisoning phenomenon above 100 °C.

In terms of the 10-Pd/ZrCo samples, the phenomenon of poisoning was similar to those of the ZrCo samples at 10 °C, 200 °C, and 300 °C. Nevertheless, the hydrogen content of the ZrCo and 10-Pd/ZrCo films exhibited a conspicuous difference after poisoning at 100 °C, among which the ZrCo film suffered from serious poisoning, while the 10-Pd/ZrCo film was less affected. Moreover, the saturated hydrogen absorption capability of 10-Pd/ZrCo descended by only 0.06 wt%, although it took as long as 1000 s to reach saturation.

**Figure 6:**
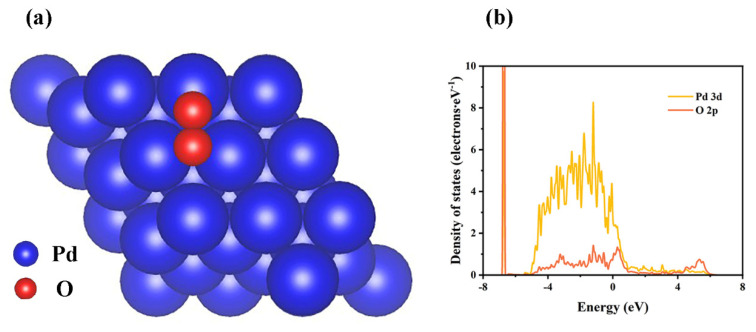
(**a**) O adsorbed on the t-f-b site of Pd (111); (**b**) PDOS distribution of the t-f-b site.

According to the SEM–EDS elemental mapping of the cross-sections of the 10-Pd/ZrCo film in Figure 4d, a certain number of O atoms converged at the bottom of the sample, indicating that the surface of the Pd layer was oxidized by the mixed H_2_ containing 1000 ppm O_2_. The oxidation of Pd can be discussed by theoretical calculation. According to the structural symmetry of Pd (111), there are only four types of adsorption of O_2_ on Pd (111). They are the top site above the atoms, the bridge site in the middle of the two top atoms, and the hollow site in the center of the three atoms on Pd (111), where the hollow site is subdivided into the FCC site and HCP site [27]. In addition, the adsorbed O_2_ on Pd (111) possesses two forms in total: upright configuration and horizontal configuration. Thus, this indicates that 12 adsorption states exist on Pd (111). The detailed calculations of the adsorption energy and O-O bond length for each state are recorded in Table S2. As a result, the adsorbed O_2_ molecules occupy the t-f-b site (t-f-b indicates that the center of mass is above the fcc hollow sites, and the two O atoms are facing the adjacent top and bridge sites), which is the most stable adsorption site on Pd (111). The adsorption energy of the site is −0.86 eV, and the O-O bond length is 1.37 Å, which is slightly higher than the bond length of the isolated O_2_ molecule (1.2 Å). Therefore, O_2_ could dissociate during adsorption on the Pd (111). To further analyze the atomic bonding of the Pd (111) and O atom, the O atom with the three nearest Pd atoms was chosen for the PDOS calculation, as indicated in Figure 6b. It is observed that the 3d orbit of the Pd atom and the 2p orbit of the O atom match one by one in the range of −6.90 eV~6.31 eV after the adsorption of the O atom. Therefore, the Pd atom has a relatively strong interaction with the O atom.

In other words, the adsorbed O_2_ molecules on Pd (111) occupy the active dissociation site to produce a small amount of oxide PdO. According to the research of Kamal Arora et al., the PdO crystal structure gradually changes to a PdH_x_ crystal structure after the passage of H_2_, so PdO exhibits hydrogenation properties similar to Pd [28]. Since the Pd layer is deposited below the ZrCo layer, it enables the partially unoxidized ZrCo to absorb H_2_ normally after O_2_ poisoning and also plays a crucial role in promoting the decomposition of H_2_ into hydrogen atoms and their rapid transport to ZrCo. Therefore, the 10-Pd/ZrCo film exhibited a more preferable hydrogenation property than that of the ZrCo film after poisoning at 100 °C. When the poisoning temperature exceeded 200 °C, the hydrogen storage capacity of the 10-Pd/ZrCo film was nearly as destroyed as that of the ZrCo film, which could be attributed to the elevated diffusion coefficient of O in both the Pd layer and ZrCo layer, leading to the formation of a large number of oxides. In a word, the combination of the Pd layer under certain conditions (e.g., hydrogen absorption temperature below 100 °C) can improve the resistance of the films to poisoning by a mixed H_2_ containing 1000 ppm O_2_.

## 4. Conclusions

The Pd, ZrCo, and Pd/ZrCo films were prepared via direct current magnetron sputtering, and the main conclusions were drawn as follows:

1. The initial hydrogen absorption kinetics were promoted by the combination of the Pd layer and the hydrogen absorption time to 95% saturation was reduced from 5490 s for the ZrCo film down to 270 s for the 10-Pd/ZrCo film.
2. The hydrogen absorption studies at 10–300 °C against O_2_ poisoning indicated that the ZrCo and 10-Pd/ZrCo films showed a deepened poisoning and a decrease in the hydrogen absorption kinetics performance with an increasing temperature. However, 10-Pd/ZrCo had an obvious enhancement of the hydrogen absorption capacity than ZrCo below 100 °C, and it could maintain 95.8% of the hydrogen storage capacity at 100 °C.
3. The adsorption energy and O-O bond length of the O_2_ molecules on ZrCo (110) were calculated as −7.46 eV and 3.55 Å, respectively, while those of O_2_ molecules on Pd (111) were −0.86 eV and 1.37 Å. O_2_ dissociates on ZrCo (110) to form ZrO_2_ easily and dissociates on Pd (111) to form a very small amount of PdO.

All results imply that 10-Pd/ZrCo film has potential applications in the ITER by virtue of the fast initial hydrogen absorption kinetics and good resistance to O_2_ gas impurities.

## Figures and Tables

**Table 1:** Sputtering parameters of the Pd, ZrCo, and Pd/ZrCo films.

Sample	Substrate	Working Pressure	Background Pressure	Sputtering Power	Deposition Time		Substrate Temperature
					Pd Layer	ZrCo Layer	
Pd	SiO_2_	0.9 Pa	2 × 10^−3^ Pa	200 W	60 min	-	500 °C
ZrCo	Si				-	60 min	
5-Pd/ZrCo	SiO_2_				5 min	60 min	
10-Pd/ZrCo	SiO_2_				10 min	60 min	
20-Pd/ZrCo	SiO_2_				20 min	60 min	

**Table 2:** The hydrogen absorption capacity, hydrogen absorption time to 95% saturation, and rate constant of the Pd, ZrCo, and Pd/ZrCo films.

	Hydrogen Absorption Capacity (wt%)	Hydrogen Absorption Time to 95% Saturation (s)	Rate Constant
Pd	0.70	24	0.402
ZrCo	1.60	5490	0.003
5-Pd/ZrCo	1.57	1596	0.009
10-Pd/ZrCo	1.55	270	0.023
20-Pd/ZrCo	1.45	124	0.049

## Data Availability

The data are contained within the article and supplementary material.

## Supplementary Materials

The following supporting information can be downloaded at: https://www.mdpi.com/article/10.3390/ma16083159/s1, Table S1: The values of the adsorption energy and the O-O bond length after adsorption of O_2_ molecules on ZrCo (110); Table S2: The values of the adsorption energy and the O-O bond length after adsorption of O_2_ molecules on Pd (111); Table S3: Thermodynamic parameters of the ZrCo-H and 10-Pd/ZrCo-H for dehydrogenation. Figure S1: XRD patterns of ZrCo film after poisoning at 100 °C; Figure S2: (a,c) Hydrogen desorption pressure-composition-temperature for ZrCo-H and 10-Pd/ZrCo-H systems; (b,d) Van’t Hoff curves for ZrCo-H and 10-Pd/ZrCo-H systems.
