# Supplementary material for: Hydrogen Absorption Performance and O2 Poisoning Resistance of Pd/ZrCo Composite Film
Source: Materials (Basel). 2023 Apr 17;16(8):3159. doi: 10.3390/ma16083159 (PMC10145738; doi:10.3390/ma16083159)
Supplement: Supplementary file 1 [file materials-16-03159-s001.zip › materials-2337739-supplementary.pdf]

**Supplementary material: Hydrogen Absorption Performance and $O_2$ Poisoning Resistance of Pd/ZrCo Composite Film**

Table S1. The values of the adsorption energy and the O-O bond length after adsorption of $O_2$ molecules on ZrCo(110).

| Adsorption site | | $\Delta E$ (eV) | $d_{O-O}$ (Å) |
|---|---|---|---|
| T1 | b | -0.70 | 1.46 |
| | c | -0.61 | 1.49 |
| T2 | c | -7.32 | 3.13 |
| B1 | a | -0.24 | 1.38 |
| | b | -1.22 | 1.46 |
| | c | -5.05 | 2.12 |
| B2 | b | -4.90 | 3.21 |
| | c | -3.25 | 2.65 |
| B3 | b | -7.46 | 3.55 |
| | c | -6.09 | 3.7 |
| H1 | b | -7.38 | 3.24 |
| | c | -5.05 | 2.12 |
| H2 | b | -7.38 | 3.24 |
| | c | -4.58 | 4.51 |

Note.

The adsorption energy of unlisted sites is calculated as positive value, and the $O_2$ molecules cannot be stably adsorbed.

Table S2. The values of the adsorption energy and the O-O bond length after adsorption of $O_2$ molecules on Pd(111).

| **Adsorption site** | $\Delta E$ **(eV)** | **$d_{O-O}$ (Å)** |
|---|---|---|
| fcc | -0.25 | 1.31 |
| hcp | -0.17 | 1.3 |
| b-t-b | -0.59 | 1.33 |
| t-f-b | -0.86 | 1.37 |
| t-h-b | -0.80 | 1.37 |
| fcc-fcc | -0.72 | 1.35 |
| hcp-hcp | -0.72 | 1.35 |
| h-b-f | -0.80 | 1.37 |
| t-b-t | -0.73 | 1.35 |
| f-t-h | -0.85 | 1.37 |

Note.

The adsorption energy of unlisted sites is calculated as positive value, and the $O_2$ molecules cannot be stably adsorbed.

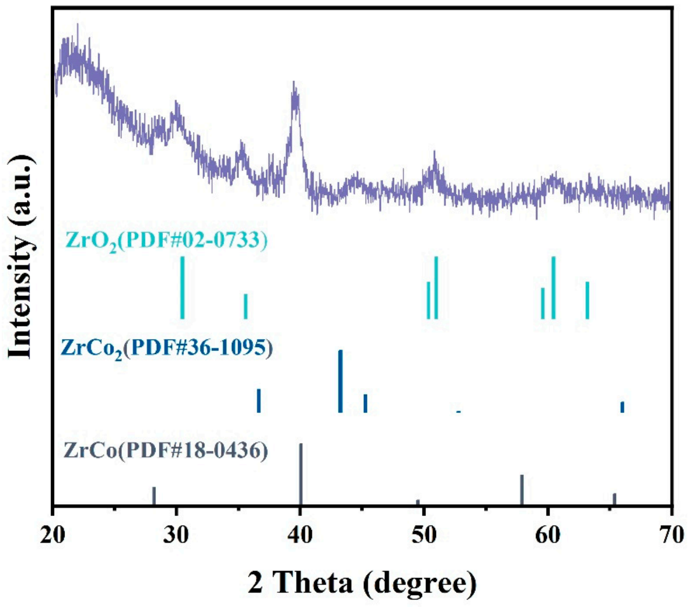


Figure S1. XRD patterns of ZrCo film after poisoning at 100 °C.

PCT (Pressure-Composition-Temperature) curve is a significant characterization to investigate the thermodynamic properties of hydrides. Figure S2. shows the PCT curves of hydrogen desorption for ZrCo-H and 10-Pd/ZrCo-H systems at 200 °C, 250 °C, 300 °C and 350 °C. Obviously, all the curves display one plateau, which indicates that only the decomposition of the $ZrCoH_3$ is presented during the hydrogen desorption in the temperature and pressure range of investigation. In order to further analyze the thermodynamic characteristics of ZrCo-H and 10-Pd/ZrCo-H systems, it is necessary to calculate the enthalpy and entropy on the basis of Van't Hoff plot:

$$ln(P) = \frac{-\Delta H}{RT} + \frac{\Delta S}{R} \tag{S1}$$

where $\Delta H$ and $\Delta S$ demonstrate the enthalpy and entropy of dissociation for hydrogen desorption reactions of ZrCo-H and 10-Pd/ZrCo-H systems, T is temperature, P denotes the plateau pressure for hydrogen desorption, R is the gas constant. Based on the temperature-dependent equilibrium plateau pressure, the relationship between $ln(P)$ vs. $1000/T$ are depicted in Figure 4. (b) (d). The $\frac{-\Delta H}{RT}$ and $\frac{\Delta S}{R}$ can be obtained from the intercept and slope of fitting linear, and the specific thermodynamic parameters of hydrides are listed in Table S3. Comparing the thermodynamic parameters of the two samples, the addition of Pd layer results in a minor reduction in the equilibrium plateau pressure at each temperature and a slight increase in the enthalpy and entropy. It is well known that the hydride stability has a great correlation with the plateau pressure, and the higher value of desorption plateau pressure, the lower the stability of hydrides. Therefore, 10-Pd/ZrCo-H system is more stable than ZrCo-H system. Furthermore, the temperature required to reach the hydrogen desorption plateau pressure of 0.1 MPa ($T_{des}$) for ZrCo-H and 10-Pd/ZrCo-H systems are calculated, as listed in Table S3. It is evident that the $T_{des}$ of the two systems is consistent relatively. The small variation of $T_{des}$ could be ascribed to the decline in the plateau and the deterioration of hydrogen absorption capacity.

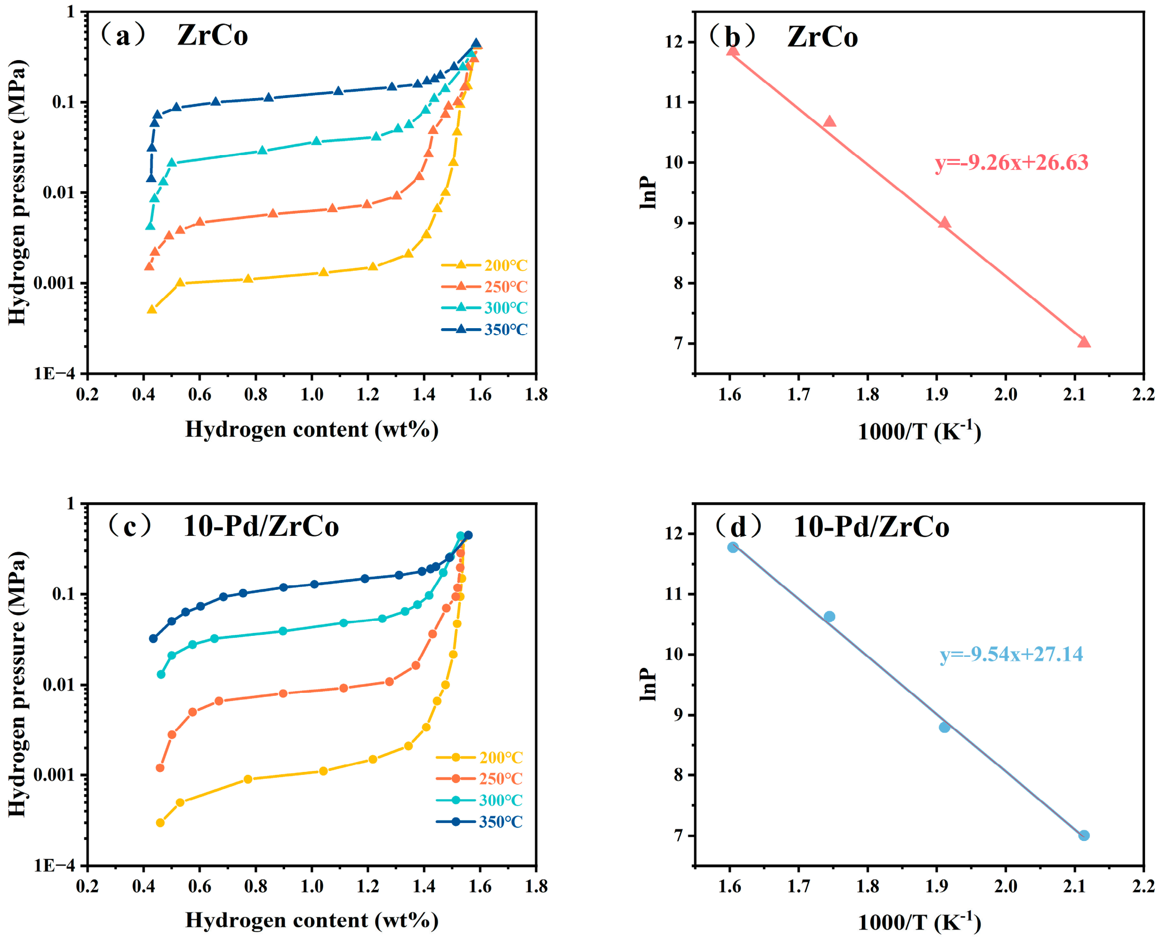


Figure S2. (a) (c)Hydrogen desorption pressure-composition-temperature for ZrCo-H and 10-Pd/ZrCo-H systems; (b) (d) Van't Hoff curves for ZrCo-H and 10-Pd/ZrCo-H systems.

Table S3. Thermodynamic parameters of the ZrCo-H and 10-Pd/ZrCo-H for dehydrogenation

| **sample** | **P[a](MPa)** | | | | **Δ*H*(kJ·mol$^{-1}$H$_2$)** | **Δ*S*(kJ·mol$^{-1}$·K$^{-1}$H$_2$)** | **Tes[b](°C)** |
|---|---|---|---|---|---|---|---|
| | **200 °C** | **250 °C** | **300 °C** | **350 °C** | | | |
| ZrCo-H | 0.0011 | 0.0080 | 0.0427 | 0.139 | 77.00 | 221.44 | 334.19 |
| 10-Pd/ZrCo-H | 0.0011 | 0.0066 | 0.0412 | 0.128 | 79.31 | 225.60 | 337.40 |

Notes.

[a] $P_{eq}$ is the equilibrium plateau pressure for hydrogen desorption.

[b] $T_{des}$ is the required temperature for hydrogen desorption plateau pressure of 0.1MPa.
